# Potential Vaccines for Treating Crohn's Disease

**DOI:** 10.29252/ibj.24.1.1

**Published:** 2019-11-01

**Authors:** Mohammad Rostami-Nejad, Mohammad Hossein Yazdi, Shekoufeh Nikfar, Ali Rezaie, Mohammad Abdollahi

**Affiliations:** 1 Gastroenterology and Liver Diseases Research Center, Research Institute for Gastroenterology and Liver Diseases, Shahid Beheshti University of Medical Sciences, Tehran, Iran;; 2 Biotechnology Research Center, Faculty of Pharmacy, Tehran University of Medical Sciences, Tehran, Iran;; 3 Pharmaceutical Sciences Research Center (PSRC), The Institute of Pharmaceutical Sciences (TIPS), Tehran University of Medical Sciences, Tehran, Iran;; 4 Department of Pharmacoeconomics and Pharmaceutical Administration, Faculty of Pharmacy, Tehran University of Medical Sciences, Tehran, Iran;; 5 Cedars-Sinai Medical Center, Los Angeles, CA, USA;; 6 Department of Toxicology and Pharmacology, Faculty of Pharmacy, Tehran University of Medical Sciences, Tehran, Iran

**Keywords:** Crohn’s disease, Inflammatory bowel diseases, Vaccines

## Abstract

CD is an inflammatory disease of the GIT and can affect several parts of the digestive system. There is a relationship between impaired mucosal barrier in the GIT of IBD patients and the role of bacteria such as *MAP* in CD. Apart from different therapeutic approaches for treating CD, development of a vaccine is a novel modality. In the present article, most available therapeutic opportunities in the last decade, especially the possibility of vaccines against CD, are reviewed. According to the search, availability of a new generation of vaccines against CD is expected specially tolerogenic *ex vivo*-derived DC-based vaccines. Regarding different locations of the challenge and the variety of clinical manifests of CD and also the type of resident antigen-presenting cells and their traffic in different parts of GIT, the results of immunotherapy with DC-based vaccines may vary case by case.

## INTRODUCTION

Most inflammatory diseases regardless of their etiology need immunosuppressive medications to control the symptoms and to inhibit the progress of inflammation. Several systemic anti-inflammatory drugs, including corticosteroids (prednisone), biological therapy (e.g. anti- TNF-α, integrin, and IL-12/23 antibodies), as well as immunosuppressive drugs such as azathioprine, methotrexate, or 6-mercaptopurine are currently available for the treatment of CD; however, many patients do not fully respond to such therapies^[^^1^^]^. These medications are associated with a significant number of short- and long-term side effects, which limit their administration. These side effects vary considerably from new-onset hyperglycemia in patients who receive glucocorticoids for their inflammation^[^^2^^]^ to drug-induced liver injury in patients with IBD treated with azathioprine and methotrexate^[^^3^^]^.

Vaccination is one of the most successful medical strategies in preventing infectious diseases. In addition to prophylactic vaccines, using vaccination for therapeutic goals has found a growing trend. So far, several therapeutic vaccines have been examined for the targeted treatment of cancer or chronic viral infections, such as HBV, HIV, as well as hepatitis B and C viruses whose infections can result in malignancies if remain untreated^[^^4^^]^. In the light of recent advances in the understanding of immune mechanisms of various inflammatory disorders and the way of resetting balance between immune effectors and regulatory cells, scientists are now able to design vaccines for patients who suffer from autoimmunity or allergic diseases^[^^5^^]^. 

DNA vaccine (plasmid DNA, when injected into the skin or muscle of mice, triggers an immune response to encoded antigens) and gene therapy (human gene transfer, which is a way to deliver a DNA construct to human genome to repair or edit a mutation) are two novel promising strategies. The current knowledge of vaccine development can be used to treat different types of inflammatory diseases, including rheumatoid arthritis, asthma, psoriasis, celiac disease, and IBD. Meanwhile, there are a number of ongoing clinical trials on vaccine and immunotherapy of rheumatoid arthritis^[^^6^^]^. Regarding the key role of various pro-inflammatory cytokines in the pathogenicity of inflammatory diseases, anti-cytokine therapy, such as IL-1 and TNF-α vaccination, along with immuno-suppressive drugs is a novel approach, which has recently brought some promises for the treatment of type 2 diabetes, systemic lupus erythematosus, and arthritis in clinical trials^[^^6^^-^^8^^]^. In the present review, we aim to consider the current available therapeutic options for the treatment of CD as one of the most common types of IBD, and to evaluate the possibility of vaccine development for the treatment of this disorder.

**CD and risk factors**

CD is one of the most common types of IBD that can affect any part of the GIT from the mouth to the anus. Symptoms and clinical manifestations of this growing disorder vary from mild to severe, including abdominal pain, diarrhea (sometimes bloody if the inflammation is severe), fever, and weight loss^[^^9^^]^. Depending on the location of this complex inflammatory disease, different clinical manifestations are expected. The location of the disease also affects the type of treatment and the clinical outcome, particularly when the immunotherapy is used. UC is another type of IBD which, in contrast to CD, can present some inflammatory manifestations only in the colon. Both of these types of IBD emerge due to several risk factors and can predispose the patients to a higher risk of developing gastrointestinal cancers^[10]^. Genetic susceptibilities and environmental components are important risk factors for CD. According to some studies, MAP, a zoonotic pathogen, seems to be the main cause of CD^[^^11^^,^^12^^]^. A meta-analysis study determined the relationship between MAP and CD, but the role of MAP in the pathogenesis of CD is yet to be clarified^[^^13^^]^. The positive effect of antibiotics in management of CD and its complications^[^^11^^-^^13^^]^ supports the involvement of bacteria in IBD. Meanwhile, the role of body immune system in CD is very important^[^^14^^,^^15^^]^. A frame shift mutation in the *NOD2* gene (*CARD15* gene) is the first linked mutation to the prevalence of CD^[^^16^^]^. So far, several genes have been found whose mutations are associated with CD, and most of these genes are known in terms of the relevant function. For instance, any point mutation in the *IL23R*, *SLC11A1*, *IRGM*, and *ATG16L1* loci will result in susceptibility to CD^[^^17^^-^^20^^]^. 

The increased incidence of CD in recent years, particularly in developing and industrialized countries, may indicate the role of environmental factors in the initiation of this inflammatory disease. Excessive consumption of meat and polyunsaturated fatty acids such as omega 6 instead of omega 3 may be associated with an increased risk of CD^[^^21^^]^. The result of different surveys has suggested that despite the usual expectation, there is no relationship between fish consumption and the incidence of CD^[^^22^^]^. Nevertheless, it is still controversial whether isotretinoin, as a vitamin A derivative used for dermatological problems, is associated with CD^[^^23^^]^. Smoking like any other health problems has a devastating role in the prognosis of CD and can cause the refractory form of the disease^[^^24^^]^. In summary, CD is a multifactorial disorder caused by a combination of genetic, environmental, immune dysregulatory and intestinal mucosal factors, including the microbiota^[25]^. 

***Role of immune regulation***

**Fig. 1:**
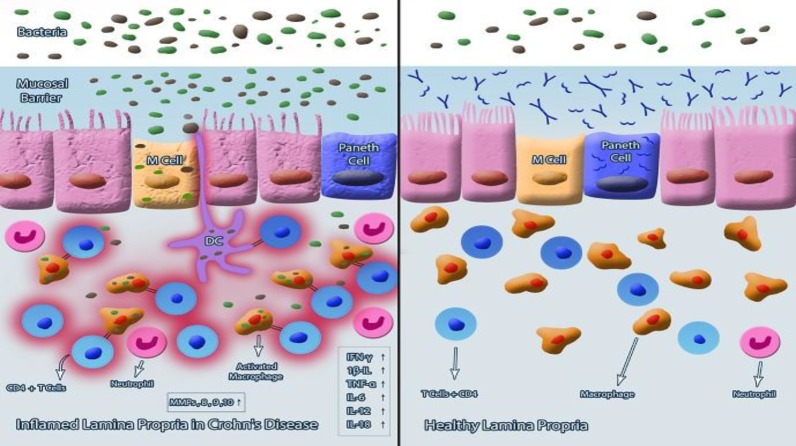
Cellular mechanism of CD and involving elements. The right picture shows the normal physiological function of mucosal barrier due to the existence of some anti-bacterial components such as α-defensin and secreted IgA and normal lamina propria. The left picture indicates the same tissue in CD in which the cascade of unfavorable inflammation is triggered by the invasion of the gut bacteria through the disturbed mucosal barrier to underlying tissues and resulting tissue damage due to the increased level of MMP and inflammation

The role of innate and adaptive immunity in pathogenesis of CD is critical. It is well accepted that UC and CD occur due to abnormalmucosal immune function and are mostly related to innate immunity, along with dysregulated or excessive Th1 cells in CD or Th2 cells in UC, which are both related to adaptive immune responses^[^^10^^]^. It was first proposed that CD is a primary T cell autoimmune disorder, though recent findings have suggested that CD can also result from an impaired innate immunity^[^^26^^]^. Innate immunity, as the first-line defense mechanism against foreign microorganisms, is responsible for the initiation of the bactericidal activity in the GIT. In the comparison between inflamed and non-inflamed colonic tissue in CD patients, it has been observed that guanosine triphosphatase signaling pathway is strongly down-regulated in non-inflamed colonic mucosa. Therefore, it has been proposed that the suppression of this pathway will help the innate immunity to exert its anti-bacterial activity, which may result in the remission of CD^[^^27^^]^. Despite the complexity of molecular mechanisms of antibacterial immunity in the GIT, clinical evidence of inadequate neutrophil infiltration, insufficient macrophage activity, and impaired pathogen clearance in CD reinforce the hypothesis that CD is a disease of innate immune dysregulation^[^^28^^]^. Indeed, intact innate immunity in healthy individuals can suppress the translocation of gut bacteria through the mucosal barrier, which may result in the triggering the acute inflammatory response (Fig. 1).

Studies have suggested that the pathogenesis of CD is related to Th1 and Th17 cell response rather than innate immunity^[^^29^^]^. Th1 has a pro-inflammatory feature and is involved in the pathogenesis of most inflammatory diseases^[^^27^^]^. Th17 is another type of CD4+ T cells, which has a bilateral role in the clearance of extracellular pathogens and also pathogenesis of several autoimmune and inflammatory diseases^[^^30^^]^. However, the recent proteomic mass spectrometry study of biopsy samples from CD patients indicated the dominance of Th1 response in these patients^[^^31^^]^. Th17 cells have the ability to shift into the Th1 phenotype, especially in the presence of IL-12 and TNF-α. These Th17-derived cells, categorized as non-classic Th1 cells, are potential targets for anti-inflammatory therapeutic goals^[^^30^^]^. The efficacy of various biological therapies have been demonstrated in CD^[^^32^^-^^36^^]^, further supporting the role of immune dysregulation in CD development. 

Modern hygiene standards due to the industrial life can affect the characteristics of immune responses. It has been hypothesized that the evolution of the immune system by exposure to bacteria and parasites can help the maturation of immunity and the ability of immune response to attack foreign microorganisms. Anti-inflammatory effects of helminths proved by several studies and the corresponding mechanism have been indicated to inhibit IFN-γ and IL-17 production as pro-inflammatory cytokines and to promote IL-4, IL-10, and TGF-β production as immune-regulatory cytokines^[^^37^^]^. 

***Role of gut microbiome***

Gut microbiota is a complex community of different microorganisms colonized in the digestive tracts of humans and other animals^[^^38^^]^. CD occurs in individuals who are exposed to some environmental factors and/or some commensal microbiota, which can disturb mucosal immune reaction and trigger an unfavorable inflammation^[^^39^^]^. The close connection between the gut microbiota and the intestinal mucosa frequency modulates and shapes the gut immune system in CD patients^[^^40^^,^^41^^]^. Several studies have shown the diminished diversity of gut microbiome in CD along with a reduction in *Bifidobacteriaceae*, *Faecalibacterium*, *Clostridiales*, and *Roseburia* plus an increase in *Enterobacteriaceae* spp., Bacteroides spp., and *Mycobacterium avium* subspecies paratuberculosis^[^^42^^-^^45^^]^. Gerasimidis *et al.*^[^^46^^]^ collected fecal samples from 15 children with CD and 21 healthy controls. They found that *Faecalibacterium prausnitzii* spp. significantly reduced after 30 days on exclusive enteral nutrition. Global microbial richness significantly reduced as compared to controls. Fujimoto *et al.*^[^^47^^]^ have also observed a significant reduction in the number of *Faecalibacterium prausnitzii* spp. in CD patients in comparison to healthy controls. The authors concluded that anti-inflammatory responses in the lumen and a decrease in the commensal microbiota may be relevant to the *F. prausnitzii* reduction. In contrast, another study on 151 fecal samples of CD patients in Japan detected no regional difference in the fecal microbiota profiles of the healthy subjects and confirmed the reduced number of *Faecalibacterium prausnitzii* spp. in CD patients compared with the controls. That study also found a significant drop in *Bacteroides* and *Bifidobacterium* during the active phase of CD^[^^48^^]^.

Previous studies have suggested that the increased incidence of CD is associated with smoking. Although smoking is not directly considered as a risk factor in CD patients, it may contribute to a dysbiosis of the gut microbiota, which is known as an important risk factor; however, the causal mechanisms of this relationship need further investigation^[^^49^^,^^50^^]^. In a recent study, the frequency of *Collinsella*, *Enterorhabdus*, and Gordonibacter in 21 smoking subjects significantly reduced in comparison with 21 nonsmoking CD patients^[^^49^^]^. In contrast, another study in 2012 reported that *Bacteroides*-*Prevotella* significantly elevated in smokers in comparison to nonsmokers and healthy controls^[^^50^^]^. In 2014, 447 (children and adolescents) newly diagnosed CD patients and 221 controls were compared. This report showed that those earlier proposed microbiota (as a biomarker for IBD) such as *Pasteurellaceae (Haemophilus sp.*),* Veillonellaceae*,* Neisseriaceae*,* Clostridiales*, and* Fusobacteriaceae* increased in patients, while the level of *Bacteroides*, *Faecalibacterium*, *Roseburia*, *Blautia*, *Ruminococcus*, and *Coprococcus* decreased^[^^45^^]^. Primary clinical improvement could be expected by microbial functional structure, preliminarily with a frailer impact at the level of species or genera.

Regarding all above studies and even some controversial evidence on the impact of gut bacteria on CD, to the best of the authors' knowledge, there is a well-accepted relationship between impaired mucosal barrier and inefficient gut microbiome, which can take a protective role in the GIT of IBD. It seems that the microorganisms in the GIT can benefit from their host's disabled mucosal layer and defect in the clearance of bacteria from the intestinal walls. 

***Current available therapies***

Previous research has proposed that CD is not a particular disease entity because of its varied nature; rather a range of related idiopathic conditions co-occur ^[^^51^^,^^52^^]^. Although this issue is highly controversial, some believe that bacterial infections such as MAP are one of the key players in the induction of idiopathic CD. The usefulness of antimicrobial therapies has long been documented for CD, but this treatment is ordered for short courses and not commonly applied within standard therapeutic regimes. Despite debatable and incompatible clinical trials reporting a long-term antimycobacterial therapy, studies are mostly focusing on immunology and gene therapy in CD^[^^53^^-^^56^^]^. CCX282-B called vercirnon was trialed among 436 CD patients who were prescribed CCX282-B or placebo for 12 weeks, then received 250 mg CCX282-B twice daily until week 16. After week 16, those with clinical improvement were also randomly divided into groups given placebo or CCX282-B for 36 weeks^[^^55^^]^. Vercirnon offers a novel approach to reduce intestine-specific inflammatory responses, but the findings of this clinical trial recommended conducting phase III clinical trials in CD. 

Regarding the role of IL-17 in pathogenesis of CD, many trials have designed to block this pro-inflammatory cytokine as a therapeutic approach to ameliorate the symptoms of the disease. However, some disappointing results have been observed in treatment of patients with moderate to severe CD using secukinumab, a monoclonal antibody against IL-17A^[^^56^^]^. This observation may indicate the complexity of CD pathogenesis, and the fact that it is not easy to treat this chronic disease by targeting only one of the potential causes of disease. A phase II clinical trial study of tofacitinib in 139 patients with moderate-to-severe active CD displayed that after 4-week administration of tofacitinib or placebo, no significant changes were observed in patients with clinical remission^[^^57^^]^.

Studies have suggested that vitamin D deficiency occurs in IBD patients; nonetheless, few surveys advocate a relationship between low vitamin D levels and disease activity^[^^58^^]^. A few numbers of clinical trials support this hypothesis only in patients with CD not with UC. Recently, the effect of 10,000 IU daily in comparison to 1000 IU daily of vitamin D3 oral supplementation was evaluated for 12 months in 18 and 16 patients with CD in remission, respectively^[^^59^^]^. The results showed that a higher dose of daily vitamin D3 significantly increased the serum 25-hydroxy-vitamin D levels, but the degrees of clinical relapse were similar between both groups. Nevertheless, regarding the immune regulatory effects of vitamin D and based on the clinical evidence of vitamin D deficiency in CD patients, supplementation with vitamin D can be considered as a way for the management of this inflammatory disease^[^^58^^]^.

In a study, the efficacy of combination therapy using adalimumab and azathioprine in 66 cases with active CD was compared with 69 CD patients without azathioprine^[^^60^^]^. The results showed that the percentage of endoscopic improvement in the first group of patients was significantly higher than the second group. However, no statistically significant difference was reported between the groups regarding the clinical improvement at week 26 in patients with active CD. In another study, children patients with CD were randomly divided to receive high-dose (93 cases) or low-dose (95 subjects) of adalimumab. The results showed that the safety risks did not increase with higher dose, but some clinical improvements were observed^[^^61^^]^. In a study in 2016, the safety and effectiveness of oral omega-3 supplementation for patients with CD was investigated^[^^62^^]^. Six patients received one bottle of the supplement daily for 28 days after one-month washout period, they started two bottles daily for 28 days. The result displayed that omega-3 was harmless and beneficial for maintaining remission in CD patients.

A significant number of CD patients ultimately need an intestinal surgery. In a multicenter, randomized trial, 297 patients with CD were evaluated by receiving infliximab or placebo to investigate the CD recurrence^[^^63^^]^. All investigated subjects underwent ileocolonic resection within 45 days before being included in the study and then randomly allocated to infliximab and placebo groups every eight weeks for 200 weeks. The result of this study showed that the clinical and endoscopic recurrences of CD in the placebo group (20% and 60%, respectively) are lower than in the infliximab group (12.9% and 30.6%, respectively) before or at week 76, which was statistically significant for the second one. The researchers have recommended that infliximab decreases endoscopic recurrence, but it has no preventive role regarding clinical recurrence after ileocolonic resection. Table 1 lists some of the recent therapeutic studies in IBD patients.

**Potential vaccines for the management of CD**

***Vaccine for inflammatory diseases***

The development of vaccines against cytokines as main players in initiation/progression of inflammation has been under focus^[^^69^^,^^70^^]^. As an example, to treat type 2 diabetes in a phase І clinical trial, IL-1β, as a key cytokine that involved in inflammatory diseases, has been targeted by hIL-1β/VLP vaccine, aiming to produce corresponding neutralizing antibodies^[^^71^^]^. In a preclinical study, for the treatment of systemic sclerosis, the modified form of mouse IL-6 peptide in conjugation with KLH has been used for both passive and active immunization of mice, which resulted in immunization and preventing the development of bleomycin-induced dermal fibrosis^[^^72^^]^. According to the substantial role of TNF-α in inflammatory response, targeting this pro-inflammatory cytokine would be associated with great advantages for any type of inflammatory illness. Immunizing the human body against TNF-α, as a potential target, is a novel approach for the induction of polyclonal anti-TNF antibodies in rheumatoid arthritis patients and has been tested in a phase II clinical trial^[^^7^^]^. Nevertheless, it should be noted that any anti-TNF- treatment, including monoclonal antibody and/or vaccine, will be a treatment option only in those individuals whose disease has emerged in response to the elevated levels of this pro-inflammatory cytokine and are not considered as non-responders to anti-TNF- therapy. Table 2 lists some of these recent works. 

***Vaccines targeting mucosal barrier***

The main goal of vaccination for CD disease is to improve the immune capacity and normalize the hyperactivated immune responses. As shown in Figure 1, the healthy mucosal barrier, as the first line of innate immune system, protects the underlying tissues and resident immune cells of the GIT against exposure to gut bacteria. In contrast, in CD, the impaired mucosal barrier not only causes exposure of gut bacteria including both pathogens and normal flora to immune cells and consequent inflammation but also stimulates immunity against food antigens and exacerbates an unwanted immune disorder. Therefore, the best approach to manage CD is to maintain the intestinal homeostasis.

***Vaccines*** ***targeting MAP***

As mentioned earlier, some researchers believed in the potential role of MAP in CD pathogenesis. MAP can infect patients through ingested milk and meat products. The bacterium can be isolated from milk products, even after milk pasteurization process^[^^73^^]^. Regarding the possible role of MAP and due to the bacterial nature of this probable cause of CD, there have been some efforts to limit the incidence of this infection and prevent subsequent CD^[^^74^^]^. Immunization against MAP may be considered as a prophylactic option. Although there is still no available data on clinical trials of this strategy (this is ongoing)^[^^75^^]^, the role of MAP in the pathophysiology of the CD is still a topic of great debate and demands more clinical studies. 

**Table 1:** Current therapeutic approaches for CD

**Drug**	**Type of intervention**	**Number of patients**		**Severity of disease**	**Clinical outcome**
Antimycobacterial therapy^[^^53^^]^	Treated with metronidazole and/or ciprofloxacin and divided into three groups according to antibiotic therapy	233 inpatients with CD		Active CD	70.6% with antibiotic combination, 72.8% with metronidazole, 69.0% with ciprofloxacin were improved, and most frequent symptoms and signs were disappeared
Vercirnon^[^^54^^]^	Half of patients received placebo, and half vercirnon 500 mg once daily or vercirnon 500 mg twice daily	608 patients were equally randomized to placebo and case groups		Moderately to severely active CD	Their data did not establish effectiveness of vercirnon as an induction therapy in these patients
Vercirnon	144 Subjects received placebo, 98 patients received CCX282-B 250 mg once daily, 97 cases 250 mg twice daily, and 97 subjects 500 mg once daily for 12 weeks. Then received 250 mg CCX282-B twice daily, open-label, through week 16. Subjects who had a clinical response at week 16 were randomly assigned to groups given placebo or CCX282-B (250 mg, twice daily) for 36 weeks	436 subjects randomized to four groups		Subjects with moderate-to-severe CD	CCX282-B, an orally administered specific CCR9 antagonist, provides a novel approach to modulating intestine-specific inflammatory responses
Tofacitinib^[^^57^^]^	Patients were randomly divided into 4 groups, including: 1 mg (n = 36), 5 mg (n = 34), or 15 mg (n = 35) tofacitinib or placebo (n = 34), twice daily for 4 weeks, at 48 centers in 12 countries	n= 139; age, ≥18 y		Patients with moderate-to-severe active CD	No significant differences in the proportion of patients with moderate-to-severe active CD who achieved clinical responses or clinical remission after 4 weeks' administration of tofacitinib or placebo twice daily
Vitamin D3^[^^59^^]^	Patients were randomly assigned to receive high-dose vitamin D3 at 10,000 IU daily compared to 1000 IU daily for 12 months	High dose = 18 cases Low dose = 16 cases		Patients with CD in remission	Higher dose of daily vitamin D3 was significantly increased the serum 25-hydroxy-vitamin D levels, but the degrees of clinical relapse were similar between both groups
Adalimumab^[^^60^^]^	Efficacy of adalimumab with and without azathioprine in patients with active CD	66 patients in monotherapy group and 69 subjects in combination group		Patients with active CD	Patients in combination group the percentage of endoscopic efficacy was significantly higher than in patients in monotherapy group, but no statistically difference was reported between the groups regarding clinical improvement at week 26.
Adalimumab^[^^61^^]^	Children patients were randomly assigned to high dose and low dose adalimumab	High dose (n = 93 cases) and low dose (n = 95 subjects)		Children patients with CD in remission	Safety risks were not increased with weekly dosing of adalimumab but clinically advantageous for children with CD
Omega-3 supplementation^[^^62^^]^	Patients in remission ingested one bottle (100 ml) of the test formulation (IMARK S®) daily for 28 days and after a one-month washout period, ingested two bottles of the formulation daily for 28 days	Six patients with CD		Patients with CD in remission	Oral capsule omega-3 supplementation would be harmless and beneficial for continuing remission in patients with CD
Infliximab^[^^63^^]^	Patients were randomly assigned (1:1) to groups given infliximab (5 mg/kg) or placebo every 8 weeks for 200 weeks to evaluate the efficacy of infliximab in preventing postoperative recurrence of CD	297 patients		CD patients undergone ileocolonic resection	Infliximab is not superior to placebo in preventing clinical recurrence after CD-related resection but reduce endoscopic recurrence
Tofacitinib^[^^64^^]^	Patients randomized to receive placebo, tofacitinib 5 or 10 mg twice daily for 8 weeks. Those achieving clinical response or remission were re-randomized to maintenance treatment with placebo, tofacitinib 5 or 10 mg twice daily for 26 weeks	180 subjects randomized to three groups		Patients with moderate-to-severe CD	At week 26 of maintenance, the percentage of patients with clinical response-100 or remission was 55.8% with tofacitinib 10 mg twice daily compared with 39.5% with tofacitinib 5 mg twice daily and 38.1% with placebo and these results showed a minor treatment effect
Ustekinumab^[^^65^^]^	Patients were randomly assigned to receive intravenous ustekinumab or placebo at week 0. During the maintenance phase, those responded patients at 6 weeks underwent a second randomization to receive subcutaneous injections of ustekinumab or placebo at weeks 8 and 16.	526 patients in phase I and 145/526 patients in phase II		Adults with moderate-to-severe CD	Patients with an initial response to ustekinumab had significantly increased rates of response and remission with ustekinumab as maintenance therapy.
Naltrexone^[^^66^^]^	Randomized patients received daily oral administration of 4.5-mg naltrexone or placebo	40 patients		Subjects with active CD	Naltrexone improves clinical and inflammatory activity of subjects with moderate to severe CD compared to placebo-treated controls.
Naltrexone^[^^67^^]^	Children were randomized to placebo or naltrexone (0.1 mg/kg) orally for 8 weeks, followed by open-labeled treatment with 8 additional weeks of naltrexone.	40 patients		Children with moderate-to-severe CD	Systemic and social quality of life improved with naltrexone treatment and Naltrexone therapy seems safe and may reduce disease activity.
Azathioprine and infliximab^[^^68^^]^	Patients were randomized to receive infliximab (standard induction and maintenance schedule) or azathioprine (2.5 mg/kg/day) for one year	22 consecutive CD patients	CD patients undergone ileocolonic resection		Infliximab was more effective than azathioprine in reducing histological, but not endoscopic and clinical recurrence after curative ileocolonic resection in "high risk" CD patients

Immunosuppressive antibiotics and anti-inflammatory monoclonal antibodies are current therapeutic medications against CD in different phases with different severities. Metronidazole and ciprofloxacin = antibiotics; vercirnon = an oral inhibitor of CCR9; CCX282-B = small-molecule antagonists targeting CCR9; tofacitinib = inhibitor of the enzyme JAK1 and JAK 3, which interferes with the JAK-STAT signaling pathway; ustekinumab = human monoclonal antibodies blocked IL-12 and IL-23, which help activate certain T-cell; naltrexone = the blockade of opioid receptors; adalimumab = human monoclonal antibodies against TNF-α; infliximab = chimeric monoclonal antibodies against TNF-α; azathioprine = an immunosuppressive medication

**Table 2:** Recent clinical and pre-clinical studies on vaccination against different pro-inflammatory cytokines in inflammatory related disorders

**Target for vaccination**	**Disease**	**Type of research**	**Results of study**
IL-1β^[^^71^^]^	Type 2 diabetes	Clinical study phase І	In 48 patients, neutralizing IL-1β-specific antibody responses was detectable after six injections with doses of 900 µg
			
mIL-6^[^^72^^]^	Systemic sclerosis	Preclinical study on mouse	Immunization with a modified form of mouse IL-6 peptide in conjugation with KLH can prevent the development of bleomycin-induced dermal fibrosis
			
TNF-α^[^^7^^]^	Rheumatoid arthritis	Phase II clinical trial	Therapeutic vaccination with TNF-α-induced dose and schedule-dependent anti-TNF antibodies in patients was well tolerated. Patients with raised levels of anti-TNF antibodies had signs of clinical improvement
			
IL-15^[^^85^^]^	Arthritis	Preclinical study in monkeys	Vaccination with IL-15 induced neutralizing antibodies to native IL-15 in non-human primates, which can be effective against inflammatory diseases like arthritis
			
IL-23 p19^[^^86^^]^	Chronic Colitis	Preclinical study on mouse	Down-regulation of the inflammatory responses in chronic murine colitis
			
hIFN-α^[^^8^^]^	Systemic lupus erythematosus	Phase І/II clinical trial	Six patients confirmed a significant correlation between neutralizing anti-IFN-α antibody titers and decrease in IFN scores compared to the baseline
			
mTNF-α^[^^87^^]^	Arthritis	Preclinical study on mouse	Vaccination with TNF-α epitope-scaffold immunogen DTNF7 plus transmembrane domain of diphtheria toxin significantly delayed the onset of CIA and reduced the incidence and clinical score
			
mVEGF-A^[^^88^^]^	Arthritis	Preclinical study on mouse	This vaccination led to the production of anti-VEGF polyclonal antibodies and had a significant anti-inflammatory effect in CIA

***Vaccines targeting DC***

DC are one of the most important players in the gut environment for maintaining the gastrointestinal hemostasis. It has been suggested that the inappropriate activation of DC may contribute to the pathogenesis of IBD^[^^76^^]^. In healthy individuals, DC are responsible for the induction of tolerogenic condition through activating Treg. These cells are involved in the pathogenicity of IBD during the onset or development of disease^[^^77^^]^. Indeed, the imbalance between Foxp3 + CD4 + Treg cells and T effector cells in the gut environment is the main cause of intolerance, as well as inflammation in IBD^[^^78^^]^. Also, Tol-DC, which are a heterogeneous group of DC, can be generated outside the patients’ body through *ex vivo* generation and maturation. When re-introduced to the patient's body, these cells have the ability to inhibit the responses of memory T cell (both Th1 and/or Th17), causing the T cells anergy and inducing Treg through which they can ameliorate the consequences of inflammatory responses. Regarding the role of DC and T cells in the induction of inflammation and resulting damage to the healthy tissues of the gut, the use of Tol-DC as a vaccine to induce tolerance in the GIT can be considered as a safe, efficient, and possible clinical approach for the treatment of CD^[^^79^^]^. Tol-DC can be an option for the treatment of both IBD and different types of autoimmune diseases, including type 1 diabetes and rheumatoid arthritis^[^^80^^,^^81^^]^. *Ex vivo* generated monocyte-derived DC have also been used as a vaccine for the treatment of different cancers. For instance, Sipuleucel-T is a market released DC-based cancer immunotherapy product for the metastatic form of prostate cancer^[^^82^^]^. Colorectal and non-small cell lung cancers are also taking advantage of DC cell-based vaccines^[^^83^^,^^84^^]^. Tol-DC for the treatment of autoimmune diseases and inflammatory disorders can be *ex vivo* generated through different protocols. Glucocorticoids, vitamin D, retinoic acid, and the cocktail of cytokines such as IL-1β, TNF-α, IL-6, and PGE2, have the ability to induce the tolerogenic form of DC from the immature precursor^[^^76^^]^. In nearly all of the mentioned diseases except CD, the location of the challenge is mostly stable; therefore, application of immunotherapeutic modalities will result in a predictable outcome. In CD, however, due to different locations of the challenge and variety of clinical manifestations of disease and also regarding the type of resident antigen-presenting cells and their traffic in different parts of GIT, the results of immunotherapy even with Tol-DC may be variable case by case. 

Regarding all the above facts and based on the known mechanisms of CD, development of an appropriate vaccine for the control of immune response in this inflammatory disease seems achievable, however, there is not any approved vaccine for the CD, and the only under trial product in is an anti-MAP vaccine. Both types of IBD are on the rise due to the existence of several risk factors, including genetic susceptibilities, environmental components, gut microbiome, immune system dysregulation, and the tendency to humoral or cellular type of immune responses. Regarding the pivotal role of immune system in the pathogenicity of IBD from the initiation to progressive or advanced stages, most therapeutic approaches for curing CD or UC have targeted immune responses to return the normal immunophysiological function of the gut. Traditionally, immunesuppressive drugs can improve the inflammatory symptoms; however, it may leave several side effects and can expose the patient to a higher risk of opportunistic infections. 

Since the last decade, the field of immunotherapy against different diseases, particularly against inflammatory-based diseases, has been constantly growing. The current knowledge about the pathogenicity and molecular mechanisms of immune-related diseases such as IBD has helped researchers to find potential therapeutic candidates for targeted and oriented therapy as well as management, control, and regulation of unfavorable outcomes of inflammation. The list of biological therapies such as anti-inflammatory monoclonal antibodies for the treatment of IBD is being updated, and they seem to be a better alternative for the treatment of CD rather than conventional immune-suppressive drugs such as azathioprine, mercaptopurine, and methotrexate.

Although bacterial infection such as MAP is considered as the main cause of idiopathic CD, the usefulness of antibacterial regimen against CD on the one hand and the lack of sufficient documents on the role of successful immunization for the prevention or treatment of CD on the other hand makes it difficult to judge the benefit of this approach. In another aspect, although CD is emerging due to the impaired mucosal immunity and the penetration of gut microbial flora or pathogenic bacteria to underlying tissues and resulting inflammation (Fig. 1), it is impossible and certainly irrational to immunize the body against normal flora, and there is no data to advocate the benefit of immunization against gut pathogenic bacteria to prevent or treat CD. Therefore, the context of vaccine against the CD is totally different in comparison to other same therapies in which vaccination may be considered as a useful treatment. 

**Fig. 2:**
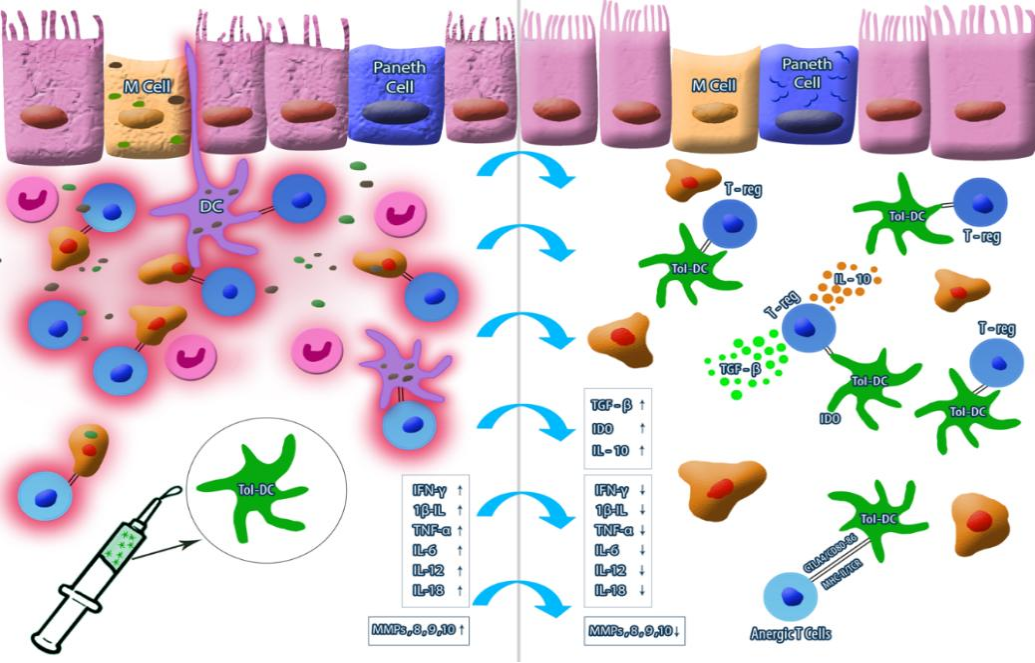
The potential of DC-based vaccine for CD. Although the impaired immune response and the lack of tolerogenic mechanism in CD patients may result in the progress of inflammatory response and deteriorate the condition, *ex vivo*-derived Tol-DC, as an immune-modulatory tool for inducing the tolerance and triggering Treg trough CTLA4-CD80-86 signaling in the intestinal microenvironment, would be considered as possible vaccine therapy against CD

Since impaired tolerance in the lamina propria of CD patients is one of the main causes of aggressiveness in the prognosis of disease, it is expected that management of this immunopathological situation and inflammation will result in clinical improvement. Although anti-TNF-αs (infliximab and adalimumab) lead to major shifts in the therapeutic paradigm of CD and are considered as promising options, several concerns on their application have still remained unresolved^[^^89^^]^. Tol-DC can help the immune system, restore tolerance by the induction of anergy or apoptosis in autoreactive T cells or stimulation of different types of Treg (Fig. 2). There are many possible mechanisms by which Tol-DC can help the re-arrangement of immune cells and repair the immunophysiological conditions in the gut microenvironment. One of the tolerogenic mechanisms to induce peripheral tolerance is the induction of type 1 Treg^[^^90^^,^^91^^]^. An increase in the level of TGF-β, as an immunosuppressive cytokine, is another related mechanism to induce peripheral tolerance in the lamina propria, which is triggered in the presence of Tol-DC^[^^92^^]^. IL-10 is another immunosuppressive cytokine produced through the interaction of Tol-DC with T cells whose role has been well demonstrated in maintaining the gut hemostasis^[^^93^^]^. 

Regarding the points discussed in this study and based on the current knowledge on the role of immune responses and factors involved in the pathogenicity of the CD and also according to the technological advances in developing vaccines for different aims, this is the time to develop a new generation of vaccines to control and suppress the unfavorable inflammatory outcomes during the acute phase of CD and return the normal immunophysiological condition in the GIT. In this scenario, Tol-DC-based vaccines, with regard to their fortune in other immunological challenges, seem to have more chance of success and could be a promising vaccine against CD in future.

## CONFLICT OF INTEREST.

 None declared.

## References

[1] Tausche AK, Richter K, Grässler A, Hänsel S, Roch B, Schröder HE (2004). Severe gouty arthritis refractory to anti-inflammatory drugs: treatment with anti-tumour necrosis factor a as a new therapeutic option. Annals of the rheumatic diseases.

[2] Suh S, Park MK (2017). Glucocorticoid-induced diabetes mellitus: An important but overlooked problem. Endocrinology and methabolism (Seoul).

[3] Tran-Minh ML, Sousa P, Maillet M, Allez M, Gornet JM (2017). Hepatic complications induced by immune-suppressants and biologics in inflammatory bowel disease. World journal of hepatology.

[4] Brezar V, Hani L, Surenaud M, Hubert A, Lacabaratz C, Lelièvre JD, Levy Y, Seddiki N (2017). Negative modulation of suppressive HIV-specific regulatory T cells by IL-2 adjuvanted therapeutic vaccine. PLoS pathogens.

[5] Wraith DC (2009). Therapeutic peptide vaccines for treatment of autoimmune diseases. Immunology letters.

[6] Benham H, Nel HJ, Law SC, Mehdi AM, Street S, Ramnoruth N, Pahau H, Lee BT, Ng J, Brunck ME, Hyde C, Trouw LA, Dudek NL, Purcell AW, O'Sullivan BJ, Connolly JE, Paul SK, Lê Cao KA, Thomas R (2015). Citrullinated peptide dendritic cell immunotherapy in HLA risk genotype-positive rheumatoid arthritis patients. Science translational medicine.

[7] Durez P, Vandepapeliere P, Miranda P, Toncheva A, Berman A, Kehler T, Mociran E, Fautrel B, Mariette X, Dhellin O, Fanget B, Ouary S, Grouard-Vogel G, Boissier MC (2014). Therapeutic vaccination with TNF-Kinoid in TNF antagonist-resistant rheumatoid arthritis: a phase II randomized, controlled clinical trial. PLoS one.

[8] Ducreux J, Houssiau FA, Vandepapelière P, Jorgensen C, Lazaro E, Spertini F, Colaone F, Roucairol C, Laborie M, Croughs T, Grouard-Vogel G, Lauwerys BR (2016). Interferon α kinoid induces neutralizing anti-interferon α antibodies that decrease the expression of interferon-induced and B cell activation associated transcripts: analysis of extended follow-up data from the interferon α kinoid phase I/II study. Rheumatology (Oxford).

[9] Kappelman MD, Moore KR, Allen JK, Cook SF (2013). Recent trends in the prevalence of Crohn’s disease and ulcerative colitis in a commercially insured U. S. population. Digestive diseases and sciences.

[10] Blumberg RS, Strober W (2001). Prospects for research in inflammatory bowel disease. JAMA.

[11] Abdolghaffari AH, Nikfar S, Rahimi HR, Abdollahi M (2012). A comprehensive review of antibiotics in clinical trials for inflammatory bowel disease. International journal of pharmacology.

[12] Nikfar S, Mirfazaelian H, Abdollahi M (2010). Efficacy and tolerability of immunoregulators and antibiotics in fistulizing crohn's disease: A systematic review and meta-analysis of placebo-controlled trials. ‎Current pharmaceutical design.

[13] Rahimi R, Nikfar S, Rezaie A (2006). A meta-analysis of broad-spectrum antibiotic therapy in patients with active Crohn's disease. ‎Clinical theaputics.

[14] Davis WC (2015). On deaf ears, Mycobacterium aviumparatuberculosis in pathogenesis Crohn's and other diseases. World journal of gastroenterology.

[15] Feller M, Huwiler K, Stephan R, Altpeter E, Shang A, Furrer H, Pfyffer GE, Jemmi T, Baumgartner A, Egger M (2007). Mycobacterium avium subspecies paratuberculosis and Crohn’s disease: a systematic review and meta-analysis. Lancet infectious diseases.

[16] Ogura Y, Bonen DK, Inohara N, Nicolae DL, Chen FF, Ramos R, Britton H, Moran T, Karaliuskas R, Duerr RH, Achkar JP, Brant SR, Bayless TM, Kirschner BS, Hanauer SB, Nuñez G, Cho JH (2001). A frameshift mutation in NOD2 associated with susceptibility to Crohn's disease. Nature.

[17] Prescott NJ, Fisher SA, Franke A, Hampe J, Onnie CM, Soars D, Bagnall R, Mirza MM, Sanderson J, Forbes A, Mansfield JC, Lewis CM, Schreiber S, Mathew CG (2007). A nonsynonymous SNP in ATG16L1 predisposes to ileal Crohn's disease and is independent of CARD15 and IBD5. Gastroenterology.

[18] Diegelmann J, Czamara D, Le Bras E, Zimmermann E, Olszak T, Bedynek A, Göke B, Franke A, Glas J, Brand S (2013). Intestinal DMBT1 expression is modulated by Crohn’s disease-associated IL23R variants and by a DMBT1 variant which influences binding of the transcription factors CREB1 and ATF-2. PLoS one.

[19] Prescott NJ, Dominy KM, Kubo M, Lewis CM, Fisher SA, Redon R, Redon R, Huang N, Stranger BE, Blaszczyk K, Hudspith B, Parkes G, Hosono N, Yamazaki K, Onnie CM, Forbes A, Dermitzakis ET, Nakamura Y, Mansfield JC, Sanderson J, Hurles ME, Roberts RG, Mathew CG (2010). Independent and population-specific association of risk variants at the IRGM locus with Crohn's disease. Human molecular genetics.

[20] Chermesh I, Azriel A, Alter-Koltunoff M, Eliakim R, Karban A, Levi BZ (2007). Crohn's disease and SLC11A1 promoter polymorphism. Digestive diseases and sciences.

[21] Shoda R, Matsueda K, Yamato S, Umeda N (1996). Epidemiologic analysis of Crohn disease in Japan: increased dietary intake of n-6 polyunsaturated fatty acids and animal protein relates to the increased incidence of Crohn disease in Japan. The American journal of clinical nutrition.

[22] Taghipour N, Aghdaei HA, Haghighi A, Mossafa N, Tabaei SJ, Rostami-Nejad M (2014). Potential treatment of inflammatory bowel disease: a review of helminths therapy. Gastroenterology and hepatology from bed to bench.

[23] Brian G Feagan, Reena Khanna (2013). Isotretinoin, acne, and Crohn’s disease: A convergence of bad skin, bad science, and bad litigation creates the perfect storm. Gastroenterology and hepatology.

[24] Cosnes J (2004). Tobacco and IBD: relevance in the understanding of disease mechanisms and clinical practice. Best practice and research: clinical gasteroentology.

[25] Regueiro M, Greer JB, Szigethy E (2017). Etiology and treatment of pain and psychosocial issues in patients with inflammatory bowel diseases. Gastroenterology.

[26] Marks DJ, Segal AW (2008). Innate immunity in inflammatory bowel disease: a disease hypothesis. Journal of pathology.

[27] Dai C, Jiang M, Sun MJ (2015). Innate immunity and adaptive immunity in Crohn’s disease. Annals of transational medicine.

[28] Marks DJ, Harbord MW, MacAllister R, Rahman FZ, Young J, Al-Lazikani B, Lees W, Novelli M, Bloom S, Segal AW (2006). Defective acute inflammation in Crohn's disease: a clinical investigation. Lancet.

[29] Elson CO, Cong Y, Weaver CT, Schoeb TR, McClanahan TK, Fick RB, Kastelein RA (2007). Monoclonal anti-interleukin 23 reverses active colitis in a T cell-mediated model in mice. Gastroenterology.

[30] Cosmi L, Liotta F, Maggi E, Romagnani S, Annunziato F (2014). Th17 and non-classic Th1 cells in chronic inflammatory disorders: two sides of the same coin. International archives of allergy and immunology.

[31] Riaz T, Sollid LM, Olsen I, de Souza GA (2016). Quantitative proteomics of gut-derived Th1 and Th1/Th17 clones reveal the presence of CD28+ NKG2D-Th1 cytotoxic CD4+ T cells. Molecular and cellular proteomics.

[32] Zaboli P, Abdollahi M, Mozaffari S, Nikfar S (2017). Tumor necrosis factor-alpha antibodies in fistulizing Crohn’s disease: an updated systematic review and meta-analysis. Journal of research in pharmacy practice.

[33] Mozaffari S, Nikfar S, Abdolghaffari AH, Abdollahi M (2014). New biologic therapeutics for ulcerative colitis and Crohn's disease. ‎Expert opinion biological therapy.

[34] Nikfar S, Ehteshami-Afshar S, Abdollahi M (2013). Is certolizumab pegol safe and effective in the treatment of patients with moderate to severe crohn's disease? A meta-analysis of controlled clinical trials. Iranian Red Crescent medicine journal.

[35] Ehteshami-Afshar S, Nikfar S, Rezaie A, Abdollahi M (2011). A systematic review and meta-analysis of the effects of infliximab on the rate of colectomy and post-operative complications in patients with inflammatory bowel disease. Archives of medical science.

[36] Rahimi R, Nikfar S, Abdollahi M (2007). Do anti-tumor necrosis factors induce response and remission in patients with acute refractory Crohn's disease? A systematic meta-analysis of controlled clinical trials. Biomedicine and pharmacotherapy.

[37] Ben-Ami Shor D, Harel M, Eliakim R, Shoenfeld Y (2013). The hygiene theory harnessing helminths and their ova to treat autoimmunity. Clinical reviews in allergy and immunology.

[38] Lepage P, Leclerc MC, Joossens M, Mondot S, Blottière HM, Raes J, Ehrlich D, Doré J (2013). A metagenomic insight into our gut's microbiome. Gut.

[39] Pascal V, Pozuelo M, Borruel N, Casellas F, Campos D, Santiago A, Martinez X, Varela E, Sarrabayrouse G, Machiels K, Vermeire S, Sokol H, Guarner F, Manichanh (2017). A microbial signature for Crohn's disease. Gut.

[40] Imhann F, Vich Vila A, Bonder MJ, Fu J, Gevers D, Visschedijk MC, Spekhorst LM, Alberts R, Franke L, van Dullemen HM, Ter Steege RWF, Huttenhower C, Dijkstra G, Xavier RJ, Festen EAM, Wijmenga C, Zhernakova A, Weersma RK (2016). Interplay of host genetics and gut microbiota underlying the onset and clinical presentation of inflammatory bowel disease. Gut.

[41] Nikfar S, Rahimi R, Rahimi F, Derakhshani S, Abdollahi M (2008). Efficacy of probiotics in irritable bowel syndrome: A meta-analysis of randomized, controlled trials. Disease of the colon and rectum.

[42] Rahimi R, Nikfar S, Rahimi F, Elahi B, Derakhshani S, Vafaie M, Abdollahi M (2008). A meta-analysis on the efficacy of probiotics for maintenance of remission and prevention of clinical and endoscopic relapse in Crohn's disease. ‎Digestive diseases and sciences.

[43] Ricanek P, Lothe SM, Frye SA, Rydning A, Vatn MH, Tønjum T (2012). Gut bacterial profile in patients newly diagnosed with treatment-naïve Crohn's disease. Clinical and experimental gastroenterology.

[44] Gevers D, Kugathasan S, Denson LA, Vázquez-Baeza Y, Van Treuren W, Ren B, Schwager E, Knights D, Song SJ, Yassour M, Morgan XC, Kostic AD, Luo C, González A, McDonald D, Haberman Y, Walters T, Baker S, Rosh J, Stephens M, Heyman M, Markowitz J, Baldassano R, Griffiths A, Sylvester F, Mack D, Kim S, Crandall W, Hyams J, Huttenhower C, Knight R, Xavier RJ (2014). The treatment-naive microbiome in new-onset Crohn's disease. Cell host and microbe.

[45] Thorkildsen LT, Nwosu FC, Avershina E, Ricanek P, Perminow G, Brackmann S, Vatn MH, Rudi K (2013). Dominant fecal microbiota in newly diagnosed untreated inflammatory bowel disease patients. Gastroenterology research and practice.

[46] Gerasimidis K, Bertz M, Hanske L, Junick J, Biskou O, Aguilera M, Garrick V, Russell RK, Blaut M, McGrogan P, Edwards CA (2014). Decline in presumptively protective gut acterial species and metabolites are paradoxically associated with disease improvement in pediatric Crohn's disease during enteral nutrition. Inflammation bowel diseases.

[47] Fujimoto T, Imaeda H, Takahashi K, Kasumi E, Bamba S, Fujiyama Y, Andoh A (2013). Decreased abundance of Faecalibacterium prausnitzii in the gut microbiota of Crohn's disease. Journal of gastroenterology and hepatology.

[48] Andoh A, Kuzuoka H, Tsujikawa T, Nakamura S, Hirai F, Suzuki Y, Matsui T, Fujiyama Y, Matsumoto T (2012). Multicenter analysis of fecal microbiota profiles in Japanese patients with Crohn's disease. Journal of gastroenterology.

[49] Opstelten JL, Plassais J, van Mil SW, Achouri E, Pichaud M, Siersema PD, Oldenburg B, Cervino AC (2016). Gut microbial diversity is reduced in smokers with Crohn's disease. Inflammatory bowel diseases.

[50] Benjamin JL, Hedin CR, Koutsoumpas A, Ng SC, McCarthy NE, Prescott NJ, Pessoa-Lopes P, Mathew CG, Sanderson J, Hart AL, Kamm MA, Knight SC, Forbes A, Stagg AJ, Lindsay JO, Whelan K (2012). Smokers with active Crohn's disease have a clinically relevant dysbiosis of the gastrointestinal microbiota. Inflammatory bowel diseases.

[51] Korzenik JR, Dieckgraefe BK (2000). Is Crohn's disease an immunodeficiency? A hypothesis suggesting possible early events in the pathogenesis of Crohn's disease. Digestive diseases and sciences.

[52] Gibson PR (1990). Current concepts in the pathogenesis of Crohn's disease. Journal of gastroenterology and hepatology.

[53] Prantera C, Berto E, Scribano ML, Falasco G (1998). Use of antibiotics in the treatment of active Crohn's disease: experience with metronidazole and ciprofloxacin. Italian journal of gastroenterology and hepatology.

[54] Feagan BG, Sandborn WJ, D'Haens G, Lee SD, Allez M, Fedorak RN, Seidler U, Vermeire S, Lawrance IC, Maroney AC, Jurgensen CH, Heath A, Chang DJ (2015). Randomised clinical trial: vercirnon, an oral CCR9 antagonist, vs placebo as induction therapy in active Crohn's disease. Alimentary pharmacology and therapeutics.

[55] Keshav S, Vaňásek T, Niv Y, Petryka R, Howaldt S, Bafutto M, Rácz I, Hetzel D, Nielsen OH, Vermeire S, Reinisch W, Karlén P, Schreiber S, Schall TJ, Bekker P (2013). Prospective Randomized Oral-Therapy Evaluation in Crohn’s Disease Trial-1 PROTECT-1 Study Group A A randomized controlled trial of the efficacy and safety of CCX282-B, an orally-administered blocker of chemokine receptor CCR9, for patients with Crohn's disease. PLoS one.

[56] Hueber W, Sands BE, Lewitzky S, Vandemeulebroecke M, Reinisch W, Higgins PD, Wehkamp J, Feagan BG, Yao MD, Karczewski M, Karczewski J, Pezous N, Bek S, Bruin G, Mellgard B, Berger C, Londei M, Bertolino AP, Tougas G, Travis SP (2012). Secukinumab in Crohn's Disease Study Group Secukinumab, a human anti-IL-17A monoclonal antibody, for moderate to severe Crohn’s disease: unexpected results of a randomised, double-blind placebo-controlled trial. Gut.

[57] Sandborn WJ, Ghosh S, Panes J, Vranic I, Wang W, Niezychowski W (2014). Study A3921043 Investigators A phase 2 study of tofacitinib an oral Janus kinase inhibitor in patients with Crohn's disease. Clinical gastroenterology and hepatology.

[58] Venkata KVR, Arora SS, Xie FL, Malik TA (2017). Impact of vitamin D on the hospitalization rate of Crohn's disease patients seen at a tertiary care center. World journal of gastroenterology.

[59] Narula N, Cooray M, Anglin R, Muqtadir Z, Narula A, Marshall JK (2017). Impact of high-dose vitamin D3 supplementation in patients with Crohn's disease in remission: A pilot randomized double-blind controlled study. Digestive diseases and sciences.

[60] Matsumoto T, Motoya S, Watanabe K, Hisamatsu T, Nakase H, Yoshimura N, Ishida T, Kato S, Nakagawa T, Esaki M, Nagahori M, Matsui T, Naito Y, Kanai T, Suzuki Y, Nojima M, Watanabe M, Hibi T; DIAMOND study group (2016). Adalimumab monotherapy and a combination with azathioprine for Crohn's disease: A prospective, randomized trial. Journal of Crohn’s and colitis.

[61] Dubinsky MC, Rosh J, Faubion WA Jr, Kierkus J, Ruemmele F, Hyams JS, Eichner S, Li Y, Huang B, Mostafa NM, Lazar A, Thakkar RB (2016). Efficacy and safety of escalation of adalimumab therapy to weekly dosing in pediatric patients with Crohn's disease. Inflammatory bowel diseases.

[62] Yasueda A, Shinzaki S, Iijima H, Mizushima T, Nishimura J, Hiyama S, Ohno S, Ito T (2016). Safety of emulsifying lipid formulation containing omega-3 polyunsaturated fatty acids for patients with Crohn's disease. Anticancer research.

[63] Regueiro M, Feagan BG, Zou B, Johanns J, Blank MA, Chevrier M, Plevy S, Popp J, Cornillie FJ, Lukas M, Danese S, Gionchetti P, Hanauer SB, Reinisch W, Sandborn WJ, Sorrentino D, Rutgeerts P; PREVENT Study Group (2016). Infliximab reduces endoscopic, but not clinical, recurrence of Crohn's disease after ileocolonic resection. Gastroenterology.

[64] Panés J, Sandborn WJ, Schreiber S, Sands BE, Vermeire S, D'Haens G, Panaccione R, Higgins PDR, Colombel JF, Feagan BG, Chan G, Moscariello M, Wang W, Niezychowski W, Marren A, Healey P, Maller E (2017). Tofacitinib for induction and maintenance therapy of Crohn's disease: results of two phase IIb randomised placebo-controlled trials. Gut.

[65] Sandborn WJ, Gasink C, Gao LL, Blank MA, Johanns J, Guzzo C, Sands BE, Hanauer SB, Targan S, Rutgeerts P, Ghosh S, de Villiers WJ, Panaccione R, Greenberg G, Schreiber S, Lichtiger S, Feagan BG; CERTIFI Study Group (2012). Ustekinumab induction and maintenance therapy in refractory Crohn's disease. The new England journal of medicine.

[66] Smith JP, Bingaman SI, Ruggiero F, Mauger DT, Mukherjee A, McGovern CO, Zagon IS (2011). Therapy with the opioid antagonist naltrexone promotes mucosal healing in active Crohn's disease: a randomized placebo-controlled trial. Digestive diseases and sciences.

[67] Smith JP, Field D, Bingaman SI, Evans R, Mauger DT (2013). Safety and tolerability of low-dose naltrexone therapy in children with moderate to severe Crohn's disease: a pilot study. Journal of clinical gastroenterology.

[68] Armuzzi A, Felice C, Papa A, Marzo M, Pugliese D, Andrisani G, Federico F, De Vitis I, Rapaccini GL, Guidi L (2013). Prevention of postoperative recurrence with azathioprine or infliximab in patients with Crohn's disease: an open-label pilot study. Journal of Crohn’s and colitis.

[69] Melmed GY, Ippoliti AF, Papadakis KA, Tran TT, Birt JL, Lee SK, Frenck RW, Targan SR, VAsiliauskas EA (2006). Patients with inflammatory bowel disease are at risk for vaccine-preventable illnesses. American journal of gastroenterology.

[70] Assier E, Bessis N, Zagury JF, Boissier MC (2017). IL-1 vaccination is suitable for treating inflammatory diseases. Frontiers in pharmacology.

[71] Cavelti-Weder C, Timper K, Seelig E, Keller C, Osranek M, Lässing U, Spohn G, Maurer P, Müller P, Jennings GT, Willers J, Saudan P, Donath MY, Bachmann MF (2016). Development of an interleukin-1β vaccine in patients with type 2 diabetes. Molecular therapy.

[72] Desallais L, Avouac J, Fréchet M, Elhai M, Ratsimandresy R, Montes M, Mouhsine H, Do H, Zagury JF, Allanore Y (2014). Targeting IL-6 by both passive or active immunization strategies prevents bleomycin-induced skin fibrosis. Arthritis research and therapy.

[73] Botsaris G, Swift BM, Slana I, Liapi M, Christodoulou M, Hatzitofi M, Christodoulou V, Rees CE (2016). Detection of viable Mycobacterium avium subspecies paratuberculosis in powdered infant formula by phage-PCR and confirmed by culture. International journal of food microbiology.

[74] Zamani S, Zali MR, Aghdaei HA, Sechi LA, Niegowska M, Caggiu E, Keshavarz R, Mosavari N, Feizabadi MM (2017). Mycobacterium avium subsp paratuberculosis and associated risk factors for inflammatory bowel disease in Iranian patients. Gut pathogens.

[75] Bannantine JP, Hines ME 2nd, Bermudez LE, Talaat AM, Sreevatsan S, Stabel JR, Chang YF, Coussens PM, Barletta RG, Davis WC, Collins DM, Gröhn YT, Kapur V (2014). A rational framework for evaluating the next generation of vaccines against Mycobacterium avium subspecies paratuberculosis. Frontiers in cellular and infection microbiology.

[76] Coombes JL, Powrie F (2008). Dendritic cells in intestinal immune regulation. Nature reviews immunology.

[77] Burzyn D, Benoist C, Mathis D (2013). Regulatory T cells in nonlymphoid tissues. Nature immunology.

[78] Mayne CG, Williams CB (2013). Induced and natural regulatory T cells in the development of inflammatory bowel disease. Inflammatory bowel diseases.

[79] Cabezón R, Benítez-Ribas D (2013). Therapeutic potential of tolerogenic dendritic cells in IBD: from animal models to clinical application. Clinical and developmental immunology.

[80] Giannoukakis N, Phillips B, Finegold D, Harnaha J, Trucco M (2011). Phase I (safety) study of autologous tolerogenic dendritic cells in type 1 diabetic patients. Diabetes care.

[81] Hilkens CM, Isaacs JD (2013). Tolerogenic dendritic cell therapy for rheumatoid arthritis: where are we now?. Clinical and experimental immunology.

[82] Plosker GL (2011). Sipuleucel-T: in metastatic castration-resistant prostate cancer. Drugs.

[83] Liu KJ, Chao TY, Chang JY, Cheng AL, Ch'ang HJ, Kao WY, Wu YC, Yu WL, Chung TR, Whang-Peng J (2016). A phase I clinical study of immunotherapy for advanced colorectal cancers using carcinoembryonic antigen-pulsed dendritic cells mixed with tetanus toxoid and subsequent IL-2 treatment. Journal of biomedical science.

[84] Giaccone G, Bazhenova LA, Nemunaitis J, Tan M, Juhász E, Ramlau R, van den Heuvel MM, Lal R, Kloecker GH, Eaton KD, Chu Q, Dunlop DJ, Jain M, Garon EB, Davis CS, Carrier E, Moses SC, Shawler DL, Fakhrai H (2015). A phase III study of belagenpumatucel-L, an allogeneic tumour cell vaccine, as maintenance therapy for non-small cell lung cancer. European journal of cancer.

[85] Rodríguez-Álvarez Y, Morera-Díaz Y, Gerónimo-Pérez H, Castro-Velazco J, Martínez-Castillo R, Puente-Pérez P, Besada-Pérez V, Hardy-Rando E, Chico-Capote A, Martinez-Cordovez K, Santos-Savio A (2016). Active immunization with human interleukin-15 induces neutralizing antibodies in non-human primates. BMC immunology.

[86] Guan Q, Burtnick HA, Qing G, Weiss CR, Ma AG, Ma Y, Warrington RJ, Peng Z (2013). Employing an IL-23 p19 vaccine to block IL-23 ameliorates chronic murine colitis. Immunotherapy.

[87] Zhang L, Wang J, Xu A, Zhong C, Lu W, Deng L, Li R (2016). A rationally designed TNF-α epitope-scaffold immunogen induces sustained antibody response and alleviates collagen-induced arthritis in mice. PLoS one.

[88] Semerano L, Duvallet E, Belmellat N, Marival N, Schall N, Monteil M, Grouard-Vogel G, Bernier E, Lecouvey M, Hlawaty H, Muller S, Boissier MC, Assier E (2016). Targeting VEGF-A with a vaccine decreases inflammation and joint destruction in experimental arthritis. Angiogenesis.

[89] Sabado RL, Balan S, Bhardwaj N (2017). Dendritic cell-based immunotherapy. Cell research.

[90] Cools N, Ponsaerts P, Van Tendeloo VF, Berneman ZN (2007). Balancing between immunity and tolerance: an interplay between dendritic cells, regulatory T cells, and effector T cells. Journal of leukocyte biology.

[91] Rutella S, Locatelli F (2011). Intestinal dendritic cells in the pathogenesis of inflammatory bowel disease. World journal of gastroenterology.

[92] Hahm K, Im Y, Parks T, Park SH, Markowitz S, Jung H, Green J, Kim S (2001). Loss of transforming growth factor β signalling in the intestine contributes to tissue injury in inflammatory bowel disease. Gut.

[93] Glocker EO, Kotlarz D, Boztug K, Gertz EM, Schäffer AA, Noyan F, Perro M, Diestelhorst J, Allroth A, Murugan D, Hätscher N, Pfeifer D, Sykora KW, Sauer M, Kreipe H, Lacher M, Nustede R, Woellner C, Baumann U, Salzer U, Koletzko S, Shah N, Segal AW, Sauerbrey A, Buderus S, Snapper SB, Grimbacher B, Klein C (2009). Inflammatory bowel disease and mutations affecting the interleukin-10 receptor. The new England journal of medicine.

